# Quantitative protein biomarker panels: a path to improved clinical practice through proteomics

**DOI:** 10.15252/emmm.202216061

**Published:** 2023-03-20

**Authors:** Johannes Hartl, Florian Kurth, Kai Kappert, David Horst, Michael Mülleder, Gunther Hartmann, Markus Ralser

**Affiliations:** ^1^ Institute of Biochemistry Charité – Universitätsmedizin Berlin Berlin Germany; ^2^ Department of Infectious Diseases and Respiratory Medicine Charité – Universitätsmedizin Berlin Berlin Germany; ^3^ Institute of Diagnostic Laboratory Medicine, Clinical Chemistry and Pathobiochemistry Charité – Universitätsmedizin Berlin Berlin Germany; ^4^ Institute of Pathology Charité – Universitätsmedizin Berlin Berlin Germany; ^5^ Core Facility‐High Throughput Mass Spectrometry Charité – Universitätsmedizin Berlin Berlin Germany; ^6^ Institute of Clinical Chemistry and Clinical Pharmacology Universitätsklinikum Bonn Bonn Germany; ^7^ Nuffield Department of Medicine The Wellcome Centre for Human Genetics Oxford UK; ^8^ Max Planck Institute for Molecular Genetics Berlin Germany

**Keywords:** Biomarkers, Proteomics

## Abstract

The utilisation of protein biomarker panels, rather than individual protein biomarkers, offers a more comprehensive representation of human physiology. It thus has the potential to improve diagnosis, prognosis and the differentiation of responders from nonresponders in the context of precision medicine. Although several proteomic techniques exist for measuring biomarker panels, the integration of proteomics into clinical practice has been limited. In this Commentary, we highlight the significance of quantitative protein biomarker panels in clinical medicine and outline the challenges that must be addressed in order to identify the most promising panels and implement them in clinical routines to realise their medical potential. Furthermore, we argue that the absolute quantification of protein panels through targeted mass spectrometric assays remains the most promising technology for translating proteomics into routine clinical applications due to its high flexibility, low sample costs, independence from affinity reagents and low entry barriers for its integration into existing laboratory workflows.

## Graphical abstract

This Commentary underscores the importance of quantitative protein biomarker panels in clinical medicine and outlines the challenges that must be addressed to identify the most promising panels and implement them into clinical routines to realise their medical potential.

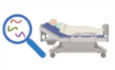

## The potential of proteomics and biomarker panel assays in medicine

**Figure 1:**
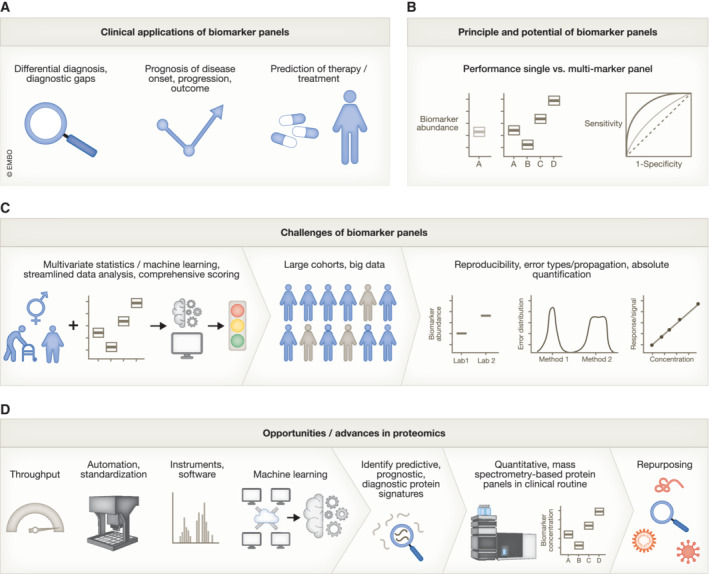
Translational multi‐marker protein panels: Current medical needs, challenges, and advancements A, BProteomics signatures and protein panel assays can fill diagnostic, prognostic and predictive gaps and improve performance compared to individual biomarkers.CAnalysis of proteomic panels together with metadata requires advanced yet streamlined data analysis pipelines and a simple scoring output for clinical interpretation. Multivariate analysis requires large cohorts to identify important protein features and build robust models. To gain sufficient data and produce reproducible protein panels, high comparability between sites and the method used to quantify markers, as well as absolute quantification are important.DRecent advances in proteomics with respect to throughput, automation, instruments and proteomics data analysis software, as well as tools to access and statistically analyse data, have facilitated the discovery of protein signatures of clinical value. Targeted mass spectrometers equipped in routine laboratories can translate these protein signatures into clinical assays. Panels or combinations of the biomarkers can be repurposed, for example in the context of infectious diseases.

The human proteome reflects physiology and pathophysiology, thus providing insights into a wide range of clinical conditions, diseases and phenotypes. Indeed, blood protein biomarkers such as troponin T, C‐reactive protein, procalcitonin and cystatin C have already established themselves as critical components of modern medicine. Proteomics describes an array of techniques aimed at analysing many proteins in parallel. In biomedicine, proteomics can be applied to characterise accessible biological fluids such as blood, urine or cerebrospinal fluid, to provide information about health and disease parameters (Geyer *et al*, 2017; Messner *et al*, 2020; Sun *et al*, 2022). Although proteomics bears the potential for improving diagnostic, prognostic and predictive tests it has not yet been fully utilized in the medical field. From a scientific point of view, this may seem surprising, given the limitations of relying solely on single or few biomarkers, particularly in diseases with diagnostic gaps, in situations with complex differential diagnoses and in patients with multiple comorbidities. By expanding from the analysis of individual protein biomarkers to protein panels or proteomes, more comprehensive prognostic tests can be developed to anticipate disease onset and progression and inform diagnostic and therapeutic decisions (Geyer *et al*, 2017; Carnielli *et al*, 2018; Messner *et al*, 2020; Xiong *et al*, 2022). In addition, proteomes and proteomic panel assays offer possibilities that exceed the potential of single biomarkers. For instance, they have better performances in predicting therapeutic success, facilitating the tailoring of specific interventions to patients or guiding treatment adjustments, e.g. in the case of therapy resistance (Fig 1A, B).

## Challenges and opportunities of implementing biomarker panels in the clinical routine

The transition from single biomarkers to the utilisation of panels or proteome signatures in clinical practice faces several technical and process‐based challenges (Fig 1C). For example, it is difficult to combine established biomarker assays into robust panel assays. The current assays are typically based on the interaction of a protein biomarker with affinity reagents, particularly antibodies, that are purchased from different providers and have batch‐to‐batch variability, with consequently poor comparability of assay results—and lack of national and international standardisation. A well‐known example is assays routinely used to determine tumour markers, which play a central role in monitoring tumour recurrence and progression (Sturgeon, 2016). Because each individual value comes with a measurement error differing from lab‐to‐lab and batch‐to‐batch, a combination of these values to form a panel would result in low robustness and reproducibility.

In order to generate panel assays that perform robustly over time and across laboratories, the analytical parameters are ideally determined by a single method that allows the quantification of errors and batch variability. Moreover, because results should be compared across laboratories and batches, absolute quantitative technologies are preferable over relative quantitative techniques. Techniques that can absolutely quantify multiple proteins out of the same sample without being dependent on affinity reagents have been spearheaded by mass spectrometry‐based proteomics. As such, the latter has established itself as a fundamental technology for basic and medical research but has long been regarded as a “too noisy” and “too slow” technology for routine clinical use. It earned this reputation as early proteomic workflows suffered from a low degree of standardisation, for example in sample preparation and data analysis, and from data acquisition methods and chromatographic regimens that provided optimal sensitivity but difficult maintenance, which lead to more sparse data and batch effects.

In recent years, this situation has improved considerably (Fig 1D). Highly standardised sample‐preparation protocols have been developed (Fu *et al*, 2018), and data‐independent acquisition techniques that provide more consistent results and fewer missing values are increasingly becoming accessible to discovery proteomics workflows (Geyer *et al*, 2017; Messner *et al*, 2020). Similarly, more sensitive and faster mass spectrometers allow proteomic workflows in conjunction with micro‐, and more and more also analytical flow rate chromatography, which is the standard in routine labs (Messner *et al*, 2020). These developments facilitate precise and routine‐compatible mass spectrometry platforms, both for discovery and for the targeted analysis of protein panels (Messner *et al*, 2020; Wang *et al*, 2022a). Furthermore, in contrast to affinity‐based techniques, mass spectrometry proteomics offers the possibility for absolute quantification through the use of stable isotope‐labelled internal standards, specifically in the targeted analysis of peptides (Pan *et al*, 2009). Absolute quantification improves statistical analysis, minimises analytical batch effects, improves cross‐study and cross‐laboratory comparability and greatly simplifies the accreditation of analytical tests. Furthermore, mass spectrometry‐based proteomics can provide high specificity and enable quantification of biomarkers where antibodies could not yet be developed—for instance in distinguishing sequence‐similar isoforms.

## Challenges and opportunities in multi‐marker panel generation

The compound scores that are typically used in clinical risk assessments are based on simple scoring systems and combine biomarkers with established physiological covariates such as body temperature, BMI and comorbidities, which make intuitive and mechanistic sense. This situation is different for multi‐marker panels that are derived from discovery proteomics data. Consequently, converting these panels into practical assays and validating their results remains more challenging. For instance, proteomic panels may consist of peptides and proteins that would not suffice as biomarkers on their own and require multi‐parametric or machine learning (ML) regression strategies in their interpretation. Moreover, the biological reasons why a certain protein value changes in a specific disease context are often not known. This situation demands a rigorous validation of marker panels. However, most clinical studies lack the sufficient size to develop robust multi‐parameter‐based regressors, given the number of variables and data dimensions a multi‐protein panel can create. Further, this brings the danger of overfitting or accidental fitting of confounders and limited generalisability to new populations (Kelly *et al*, 2019). These factors also complicate the legal frameworks required for the use of panel assays and advanced data analysis techniques—in addition to the need for new reimbursement strategies. This particularly applies when ML‐based algorithms are used beyond e.g. feature selection, to advise actual clinical decisions. The FDA has approved the first ML‐based algorithms, but key challenges remain, e.g. with respect to ethics and privacy, comparability of different algorithms and the use of continuous learning algorithms (Kelly *et al*, 2019; Warnat‐Herresthal *et al*, 2021).

Considering these challenges, it is not surprising that there are few success stories to date where proteomics‐based biomarker panels have already made their way from discovery to clinical application (Geyer *et al*, 2017). However, this situation might be about to change. The SARS‐CoV‐2 pandemic allowed moving more rapidly with the development of proteomic marker panels and bringing them closer to clinical application. For instance, we and others have shown that plasma proteomes outperform established risk assessment scores in severe COVID‐19. Indeed, proteomics was able to predict the outcome among severely ill individuals who showed similar clinical presentation (Demichev *et al*, 2022). We used discovery proteomics to generate a panel assay composed of up to 50 selected peptides that are measured on routine lab equipment. The panel robustly classified patients also in follow‐up studies, despite the changed pandemic situation (Wang *et al*, 2022a). Meanwhile, we have implemented the marker panel in a routine clinical workflow. With respect to COVID‐19, also new clinical needs have emerged. For instance, a recent study has demonstrated the potential to predict post‐COVID syndrome using plasma proteomes (Captur *et al*, 2022). Furthermore, the comprehensiveness of the panels generates a previously untapped opportunity to repurpose panel assays for new applications. For instance, the COVID‐19 panel assay, capturing many protein markers of the innate immune system, also classified monkeypox cases, despite a very different clinical manifestation of the two very different viral infections (Wang *et al*, 2022b). The repurposing of a panel to a new clinical application, such as a new pathogen, may in many cases involve only the adaptation of the statistical models used to analyse and interpret the data, but not require a validation of the laboratory part of the assay, greatly accelerating clinical implementation and regulatory procedures. Proteomic panel assays could hence greatly improve pandemic preparedness.

On the discovery end, new opportunities emerge from the availability of proteomics techniques that can capture thousands of individual proteomes and thus create population baselines (Messner *et al*, 2020; Sun *et al*, 2022). It becomes further possible to bundle multiple studies for comparative proteomic studies. This can be facilitated, for instance, through the integration of ML with blockchain technologies and swarm learning. Newly developed ML strategies can simplify collaborative big‐data studies, which comply with local data privacy regulations by sharing insights from data analysis performed at each site, but not the actual data (Warnat‐Herresthal *et al*, 2021). Here, ML can be used for automated scaling and uncovering the molecular patterns in large data sets such as proteomics. Leveraging the full potential of ML algorithms for proteomics‐based disease classification or stratification bears great diagnostic potential. The future for comparative analysis of proteome data obtained under different conditions (e.g. sample preparation, other instruments, different clinical annotation of data sets) will likely be a collaborative task in which every participating site is a node in the swarm network, participating in the model training with its local data. New nodes can enter the swarm network via blockchain smart contracts, overcoming limitations for collaborative clinical proteomics as several clinical sites may join forces to tackle the same question. As a result, new ML techniques can enable the development of algorithms that reliably and automatically extract valuable diagnostic information from non‐homogeneous proteomic data sets obtained at different sites.

## Challenges and opportunities in the clinical validation and rollout of proteomic marker panels

Once respective biomarker signatures are identified, they need to be clinically validated in order to be approved for routine use for specific indications. Here, integration of further clinical, epidemiological and laboratory data, such as defined patient diseases, BMI, sex, blood cell counts and protein and non‐protein‐based laboratory findings will be key. Simple implementation into existing sample flows of the routine laboratories is necessary for facilitating the above. Triple quadrupole mass spectrometers are used in clinical routines to quantify small molecules in newborn screening, and metabolomics profiles are now advancing towards clinical application (Kirwan, 2023). Similarly, these instruments can be used for targeted proteomics applications to measure proteomic marker panels as the latest generations of these instruments reach the level of sensitivity required for the measurement of panel assays, even if coupled with analytical flow rate chromatography that is typically applied in diagnostic laboratories. The broad availability of such platforms enables improved comparability of measurement results from different laboratories and national/international standardisation of analyte measurement, which pairs well with the increasing number of commercial sample‐preparation solutions for proteomics.

In parallel, the introduction of new technologies into routine workflows creates financial and regulatory hurdles. For instance, combining multiple analytes in a single technology means that, in certain cases, more expensive assays need to be used for parameters for which cheaper assays are already clinically approved. Indeed, many of the clinically successful biomarker assays are inexpensive and come at a cost of cents per sample. In our current regulatory framework, where each test is approved individually, each analyte is expected to provide a substantial added clinical value. Moreover, entry barriers for assays need to be low for clinical laboratories to adopt a certain technique. Instruments and software must be simple, robust and reliable, so that they can be implemented into routine workflows.

What else may help to ultimately push proteomics and multi‐marker panels into the clinical routine? First, more clinically‐oriented proteomics research studies are urgently needed. The number of reports where proteomics data sets provide a clear advantage over an existing test is increasing but is, in absolute terms, still low. With the increasing availability of proteomics‐based large data sets in conjunction with ML regression we anticipate that this situation is about to change. Second, we still lack routine‐applicable analysis and software solutions to transform the output of a proteomic measurement into an easy‐to‐use decision tool for physicians. Currently, the field is moving fast in all these directions, with facilitated access of medical research to proteomics technologies and with the deployment of several transformative technologies. We believe that these developments together will aid physicians, healthcare systems and payers to embrace clinical proteomics for routine rollouts, ultimately improving patient care.

## Disclosure and competing interests statement

Markus Ralser is a co‐founder and shareholder and Michael Mülleder a consultant of Eliptica Ltd.
